# Viral taxonomy needs a spring clean; its exploration era is over

**DOI:** 10.1186/1743-422X-10-254

**Published:** 2013-08-09

**Authors:** Adrian J Gibbs

**Affiliations:** 1 Australian National University Emeritus Faculty, Canberra ACT 0200, Australia

## Abstract

The International Committee on Taxonomy of Viruses has recently changed its approved definition of a viral species, and also discontinued work on its database of virus descriptions. These events indicate that the exploration era of viral taxonomy has ended; over the past century the principles of viral taxonomy have been established, the tools for phylogenetic inference invented, and the ultimate discriminatory data required for taxonomy, namely gene sequences, are now readily available. Further changes would make viral taxonomy more informative. First, the status of a ‘taxonomic species’ with an italicized name should only be given to viruses that are specifically linked with a single ‘type genomic sequence’ like those in the NCBI Reference Sequence Database. Secondly all approved taxa should be predominately monophyletic, and uninformative higher taxa disendorsed. These are ‘quality assurance’ measures and would improve the value of viral nomenclature to its users. The ICTV should also promote the use of a public database, such as Wikipedia, to replace the ICTV database as a store of the primary metadata of individual viruses, and should publish abstracts of the ICTV Reports in that database, so that they are ‘Open Access’.

## Introduction

The International Committee on Taxonomy of Viruses (ICTV) has recently voted to modernize and clarify its official definition of a viral species [1]. It has also suspended, at least temporarily, efforts to maintain and update the ICTV database (ICTVdB) of descriptions of individual viruses (M.J. Adams, personal communication). These two events need to be widely discussed by virologists together with any other changes that would better equip virus taxonomy for the future. Further changes are required to make the ICTV’s work more useful for a wider group of scientists than just the *cognoscenti* of viral taxonomy, especially as taxonomy is a key component of society’s response to emerging viruses. Here I discuss additional changes that would assist future progress.

## Viruses: from mysteries to muniments?

Viruses were first discovered over a century ago as intriguing pathogens whose infectivity could pass through bacteria-proof filters. Even the earliest observations showed that there are many different viruses, and initially they were distinguished by their biological characters; the hosts they infected, the symptoms they caused and the ways in which they spread from host to host. Their further characterization depended on the biochemical and biophysical inventions of the past century. When these were applied to virions, they produced groupings of viruses that shared other features [2,3], and a viral classification gradually emerged. The components of the virions were studied in greater and greater detail until the sequences of, first, viral proteins [4,5], and then viral genomes [6,7] were determined. So now, after a century groping through a fog generated by incomplete data, much of it from surrogates, the classification of known viruses is probably more complete than that of known cellular organisms, because, for the past decade, the complete genomic sequences of even the largest viruses are routinely determined. Each genomic sequence provides the complete genetic characterization of an individual viral isolate. Genomic sequences are therefore, in essence, genetic muniments, namely “documents kept as evidence of rights or privileges; archives “ (Concise Oxford Dictionary: 7^th^ Edition, 1987). Most of the phenotypic information encoded by these sequences can only be determined *in vivo* and in nature, however comparisons of gene sequences, and the proteins they encode, provide stable estimates of phylogenetic relatedness and past phylogenetic history, and so provide most of the information required for taxonomy.

## Viral names; names of what?

The first descriptions of viruses used vernacular names for viruses that were based on the diseases they caused; tobacco mosaic virus, smallpox virus, etc. In the 1930-40s, F.O. Holmes and H.H. McKinney attempted to introduce Latinized binomials for viruses, *Marmor tabaci* for TMV, however they were not adopted because the nature of viruses was still largely unknown and no sensible classification had yet emerged. Consequently throughout the past century the different host-specific branches of virology have devised their own vernacular names; plant and animal viruses from disease, host or provenance names, bacteriophages from isolate codes, etc. It is interesting, therefore, that virologists studying the Pandoraviruses, one of the currently emerging areas of virus discovery, are using classical latinized binomials for them [8].

The ICTV regularized the naming of viruses in 1993 [9], and produced a list of approved taxonomic names for 3,600 of them. Confusingly most of these were already in use as vernacular names of viruses, but they were distinguished by writing the vernacular name of the virus in Roman script and the formal name of the species in italics [10]. Thus *Tobacco mosaic virus* became the name of the species that includes the sub-cellular organism named tobacco mosaic virus (TMV). This pedantic nomenclatural practice is frequently used incorrectly or ignored, and also conflicts with the frequent use of generic or specific names of plants as part of a viral name [11]; the long-established taxonomies of the larger cellular organisms take a more relaxed view of taxonomic and organismal names, and rely more on the good sense of the reader to infer the status of a name from the context in which it is used.

A very significant unresolved problem with the taxonomic names of viruses published by the ICTV is that there is no formal description of the virus to which each name is attached. By contrast, the formal taxonomic name for each plant, animal or bacterium is attached to one particular specimen, culture, description, or even illustration, kept in a secure collection, museum or herbarium (http://en.wikipedia.org/wiki/Type_(biology). These are called ‘type’ specimens and each is permanently associated with a particular name, and thus provides total stability to that name. These preserved types can be compared with new specimens, and can be examined by techniques that have been invented since the type was collected, most notably gene sequencing. By contrast there are no comparable viral types; specimens or descriptions. In the early days of viral taxonomy most viruses could not be stored or maintained by serial passaging. Attempts were therefore made to collate viral descriptions based on the best data winnowed from the scientific literature. The earliest compilations were books, such as Smith’s “Textbook of Plant Virus Diseases” (1957) and Andrewes and Pereira’s “Viruses of Vertebrates” (1978). The first computer database of descriptions of viruses was started in the early 1980s as the VIDE (Virus Identification Data Exchange) project [12] using the pioneering DELTA system (http://delta-intkey.com/www/refs.htm). It aimed to be comprehensive and progressive, and in 1991 became the first component of the ICTVdB [13], which, when work on it was concluded in 2008, contained data on 4949 ‘species’, 286 ‘genera’ or groups and 71 ‘families’, etc. of viruses. However the descriptions in the ICTVdB never became the basis of the formal names as none uniquely described individual viruses, and all were only ever partially complete as the exploratory phase of virology moved ever forward using ever more sophisticated techniques. Nowadays viruses are identified by gene sequence comparisons using the Genbank/EMBL/ DDBJ nucleotide databases, which currently contain over two million viral gene sequences, many of them complete. Therefore unique ‘type genomic sequences’ could and should now be formally linked with the approved names of individual virus species. This was done informally in the 9^th^ ICTV Report [14] using the NCBI Reference Sequence Database [15], as this is a “comprehensive, integrated, non-redundant set of reference sequences”. Work on the ICTVdB is unlikely to be resumed as its proposed functions in viral taxonomy have been largely superseded by genomic sequences.

## Viral classification; polyphyletic or pseudo-monophyletic?

The recently approved concept of a viral species, like its predecessor, states that viral species are produced by divergent evolution; a species is now “a monophyletic group of viruses” and, earlier, “a replicating lineage”. However a feature of the ICTV’s Master Species List [16], that is cryptic and may confuse many, is that whereas the lineages that constitute Species and Genera are collections of viruses that share most of their genes, many viral Families and Orders share their genes with more than one Family or Order. Thus the Master Species List is arranged as a series of separate hierarchies, but whereas some are mostly ‘natural’, others are ‘artificial’; polyphyly masquerading as monophyly.

If, for example, a virus is stated to be from the Order *Tymovirales*, that information only reveals which gene family supplied its replicase, although if that virus was stated to be from the *Tymoviridae* or the *Alphaflexiviridae*, namely Families in the Order, that would be much more informative as two or more genes in the viruses of each Family are related, and are expressed in a range of shared phenotypic characters. Thus there is no informational gain from linking these two Families into an Order based on a single character. Similarly many species of the *Caudovirales* share no genes with other species of the Order, but have virions of similar shape, which presumably reflects their mode of infection, therefore it would be better that the formal name of this polphyletic group be abandoned, and for the traditional vernacular name ‘tailed phages’ to be used when required!

## Conclusions; *carpe diem*

Now is the time for the ICTV to review the results of its considerable efforts over the past half century, and to remove any unhelpful vestiges of that period.

Firstly its Master Species List should be ‘spring-cleaned’. The only viruses to be given approved (italicized) names should be those formally linked with a single properly documented ‘type genomic sequence’. Others that have partial sequences should have the status of ‘tentative species’. All others should be removed from the list. In the 9^th^ ICTV Report, for example, the genus *Potyvirus* had 143 approved species, yet only 53 of these were listed with NCBI Reference Sequence Accession Codes, and 16 had no sequence data. This change would indicate to a knowledgeable user the informational status of a taxonomic name, and give some idea of how fully the virus has been characterized. These sequences would also provide a series of agreed ‘datum points’ from which sequence relationships of other isolates and viruses could be measured.

Second the ICTV should only recognise and list taxa that are predominantly monophyletic and, where possible, this status should be based on sequence and structural comparisons of viral molecules. The ICTV could produce listings and phylograms, not only of viral species and higher monophyletic groupings of those species, but also lists and phylograms of the genes shared by different polyphyletic viral taxa and cellular organisms [17-19]. Listings of this sort would draw attention to the phylogenetic relationships of both the viruses and of their genes, and would therefore promote a wider understanding of the true polyphyletic origins of viruses.

Third the ICTV should not resume work on the ICTVdB and instead promote the storage of metadata of viruses in public databases, such as Wikipedia (http://en.wikipedia.org/wiki/Main_Page) or the Encyclopedia of Life (http://www.eol.org). There are already some excellent virus descriptions in Wikipedia (http://en.wikipedia.org/wiki/Tobacco_mosaic_virus, http://en.wikipedia.org/wiki/Poliovirus and http://en.wikipedia.org/wiki/Celery_mosaic_virus ), and these have been transcribed into the Encyclopedia of Life. Furthermore the remaining useful data from the ICTVdB should be transcribed into public database descriptions.

Virologists have made a considerable effort over the past century to record and interpret the variability of viruses. The viral classifications published in the ICTV Reports are a significant part of that record. They are an important resource for those identifying and describing novel and emerging viruses. This information has come, mostly *pro bono*, from a wide variety of sources. The primary metadata, together with abstracts of the information extracted from it by the ICTV and published in its Reports, must be archived in a way that ensures its long term survival, while ensuring that is available to all who wish to use it. This will only be achieved if it is stored in one of the large international ‘Open Access’ databases, as these are probably less susceptible to capricious changes in funding priorities than smaller specialized databases, and because they will promote the decentralized collation, revision and enrichment of the data.

## Competing interests

The author declares that he has no competing interests.

## References

[B1] Adams MJ Lefkowitz EJ King AMQ Carstens EB Recently-agreed changes to the International Code of Virus Classification and Nomenclature Arch Virol 2013 10.1007/s00705-013-1749-9 23836393

[B2] Horne RW Wildy P An historical account of the development and applications of the negative staining technique to the electron microscopy of viruses J Microsc 1979 10 1 103 22 10.1111/j.1365-2818.1979.tb00234.x 90730

[B3] Brandes J Wetter C Classification of elongated plant viruses on the basis of particle morphology Virology 1959 10 99 115 10.1016/0042-6822(59)90022-4 13669326

[B4] Anderer FA Preparation and properties of an artificial antigen immunologically related to tobacco mosaic virus Biochim Biophys Acta 1963 10 246 8 14012745 10.1016/0006-3002(63)91077-1

[B5] Tsugita A The proteins of mutants of TMV: classification of spontaneous and chemically evoked strains J Mol Biol 1962 10 293 300 10.1016/S0022-2836(62)80073-4 13994526

[B6] Sanger F Air GM Barrell BG Brown NL Coulson AR Fiddes CA Hutchison CA Slocombe PM Smith M Nucleotide sequence of bacteriophage phi X174 DNA Nature 1977 10 687 95 10.1038/265687a0 870828

[B7] Goelet P Lomonossoff GP Butler PJ Akam ME Gait MJ Karn J Nucleotide sequence of tobacco mosaic virus RNA Proc Natl Acad Sci U S A 1982 10 5818 22 10.1073/pnas.79.19.5818 6964389 PMC347001

[B8] Philippe N Legendre M Doutre G Coute Y Poirot O Lescot M Arslan D Seltzer V Bertaux L Bruley C Garin J Claverie J-M Abergel C Pandoraviruses: Amoeba viruses with genomes up to 2.5 Mb reaching that of parasitic eukaryotes Science 2013 10 281 6 10.1126/science.1239181 23869018

[B9] Murphy FA Fauquet CM Mayo MA Jarvis AW Ghabrial SA Summers MD Martelli GP Bishop DHL Sixth Report on the International Committee on Taxonomy of Viruses 1995 Wien, New York: Springer Verlag

[B10] Van Regenmortel MHV On the relative merits of italics, Latin and binomial nomenclature in virus taxonomy Arch Virol 2000 10 433 41 10.1007/s007050050036 10752566 PMC7087156

[B11] Bos L Coming to grips with the naming of viruses; continuing discord, or a way out? Arch Virol 2007 10 649 53 10.1007/s00705-006-0919-4 17216136

[B12] Boswell KF Dallwitz MJ Gibbs AJ Watson L The VIDE (Virus Identification Data Exchange) project: a data bank for plant viruses Rev Plant Pathol 1986 10 221 231

[B13] Buechen-Osmond C Dallwitz M Towards a universal virus database — progress in the ICTVdB Arch Virol 1996 10 392 399 10.1007/BF01718409 8634029

[B14] King AMQ, Lefkowitz E, Adams MJ, Carstens EB Ninth Report of the International Committee on Taxonomy of Viruses 2012 San Diego: Elsevier Academic Press

[B15] Pruitt KD Tatusova T Maglott DR NCBI Reference Sequence (RefSeq): a curated non-redundant sequence database of genomes, transcripts and proteins Nucleic Acids Res 2005 10 Database issue D501 4 15608248 10.1093/nar/gki025 PMC539979

[B16] Anonymous 2012 http://talk.ictvonline.org/files/ictv_documents/m/msl/4090.aspx

[B17] Boyle DB Gibbs AJ Seigman LJ Both GW Coupar BE Fowlpox virus thymidine kinase: nucleotide sequence and relationships to other thymidine kinases Virology 1987 10 2 355 365 10.1016/0042-6822(87)90415-6 3027984

[B18] Lopez-Otin C Blasco R Vinuela E Munoz M Simon-Mateo C Bockamp EO Sequence and evolutionary relationships of African swine fever virus thymidine kinase Virology 1990 10 301 304 10.1016/0042-6822(90)90409-K 2389555 PMC9534224

[B19] Yutin N Raoult D Koonin EV Virophages, polintons, and transpovirons: a complex evolutionary network of diverse selfish genetic elements with different reproduction strategies Virol J 2013 10 158 10.1186/1743-422X-10-158 23701946 PMC3671162

